# Markers of muscarinic deficit for individualized treatment in schizophrenia

**DOI:** 10.3389/fpsyt.2022.1100030

**Published:** 2023-01-09

**Authors:** Heiner Stuke

**Affiliations:** ^1^Department of Psychiatry and Psychotherapy, Charité—Universitätsmedizin Berlin, Berlin, Germany; ^2^Berlin Institute of Health at Charité—Universitätsmedizin Berlin, BIH Biomedical Innovation Academy, Berlin, Germany

**Keywords:** schizophrenia, psychosis, muscarinic receptor, personalized medicine, acetylcholine, antipsychotics, biomarker

## Abstract

Recent clinical studies have shown that agonists at muscarinic acetylcholine receptors effectively reduce schizophrenia symptoms. It is thus conceivable that, for the first time, a second substance class of procholinergic antipsychotics could become established alongside the usual antidopaminergic antipsychotics. In addition, various basic science studies suggest that there may be a subgroup of schizophrenia in which hypofunction of muscarinic acetylcholine receptors is of etiological importance. This could represent a major opportunity for individualized treatment of schizophrenia if markers can be identified that predict response to procholinergic vs. antidopaminergic interventions. In this perspective, non-response to antidopaminergic antipsychotics, specific symptom patterns like visual hallucinations and strong disorganization, the presence of antimuscarinic antibodies, ERP markers such as mismatch negativity, and radiotracers are presented as possible *in vivo* markers of muscarinic deficit and thus potentially of response to procholinergic therapeutics. Finally, open questions and further research steps are outlined.

## 1. Introduction

For many decades, the dopamine theory has been the most influential neurobiological concept in understanding psychosis (1) and it is argued that all currently used antipsychotics work through directly or indirectly changing dopaminergic neurotransmission (2). Although there is converging evidence from different lines of research for the involvement of dopamine in the emergence of psychotic symptoms (1), alterations in other neurotransmitter systems have also been demonstrated, and there is growing agreement that different neurobiological etiologies can underlie psychotic syndromes (3–5).

Firstly, a glutamate theory of schizophrenia has been established mainly based on the observation that antagonists at the glutamatergic NMDA receptor, such as ketamine, phencyclidine, or endogenously formed anti-NMDA antibodies, directly and reliably cause psychotic symptoms and genetic studies showing an increased risk of schizophrenia in individuals with gene variants affecting the glutamate system (6, 7). However, since clinical trials of glutamatergic agents developed based on this theory have yielded only mixed results, no primary glutamatergic drug is to date available for routine clinical use. Currently, studies on glutamatergic drugs focus on compounds targeting specific receptor subtypes as well as on subgroup analyses to identify patient groups with marked glutamatergic alterations and hence potentially increased response to glutamatergic interventions (6, 7).

Secondly, reduced neurotransmission at acetylcholine receptors has been proposed as a non-dopaminergic mechanism underlying schizophrenia symptoms. Acetylcholine receptors are generally divided into nicotinic and muscarinic receptors according to binding ligand, and both have been implicated in the etiology of schizophrenia. Based on the extremely high rate of nicotine use in patients with schizophrenia, which has been interpreted as self-medication of existing deficits, nicotinic acetylcholine receptors, particularly the α7-subtype, have initially become the focus of research interest (8, 9). An important line of research in this context involves sensory gating, i.e., the inhibition of electrophysiological brain responses to (mostly auditory) stimuli played at close temporal intervals after an initial stimulus. Deficits in sensory gating have been frequently reported in schizophrenia patients and linked to attentional deficits (8, 10, 11). Interestingly, agonists at nicotinic receptors (e.g., nicotine) were shown to improve these sensory gating deficits, as well as some of the cognitive deficits, in schizophrenia patients (9, 12, 13). Moreover, post-mortem studies confirmed a reduced density of α7—receptors in different brain regions in deceased schizophrenia patients. Both findings renewed interest in developing nicotinic drugs for schizophrenia, especially for its cognitive deficits, which are insufficiently ameliorated by conventional antipsychotics (8, 9). However, despite promising results in preclinical and phase 1/2 trials, phase 3 trials on nicotinic agonists in schizophrenia have been disappointing due to lack of efficacy and/or tolerability and no primarily nicotinic drug is approved for schizophrenia treatment (12, 13). Current research on nicotinic agents for schizophrenia hence focuses on finding optimal dosage regimes and investigating allosteric modulators (13).

A deficit at muscarinic acetylcholine receptors in at least a proportion of patients with schizophrenia is suggested, for example, by post-mortem studies showing reduced density of muscarinic receptors in patients, by imaging studies with radioligands showing reduced availability of muscarinic receptors, and by the ability of muscarinic antagonists such as scopolamine to elicit schizophrenia-like symptoms in healthy individuals (14, 15). Based on post-mortem studies, it was proposed that approximately one quarter of schizophrenia patients belongs to a subgroup referred to as muscarinic receptor deficit sub-group with schizophrenia (MRDS), which shows a massive and widespread reduction in the density of muscarinic receptors in the CNS (16–18). Based on that, agonists and positive allosteric modulators (PAM) at muscarinic receptors have been investigated as potential schizophrenia treatments [Table 1 for an overview, (22) for further discussion]. Intriguingly, recent successes were reported in phase 2 (20) and phase 3 (21) trials of xanomeline, an agonist at the muscarinic M1 and M4 cholinergic receptors with no direct dopaminergic effect. It should be noted that in these trials, xanomeline was administered combined with trospium, a peripheral muscarinic antagonist, in order to mitigate peripheral cholinergic side effects, but for reasons of simplicity, I will speak of xanomeline as the centrally active compound in the following.

**Table 1:** Ongoing and completed trials of clinical efficacy of muscarinergic drugs in schizophrenia.

**Clinicaltrials ID**	**Drug**	**Phase**	**Patients**	**Primary efficacy outcome**	**Results/current status**
NCT04136873	Emraclidine 20 mg Emraclidine 30 mg	1b	Schizophrenia (acute exacerbation)	Change from baseline at week 6 in the PANSS total score	Significant difference of 11.9 PANSS total score points compared to placebo (pooled for both doses) (19)
NCT05227703	Emraclidine 15 mg Emraclidine 30 mg	2	Schizophrenia (acute exacerbation)	Change from baseline at week 6 in the PANSS total score	Ongoing, estimated study completion June 2024
NCT05227690	Emraclidine 10 mg Emraclidine 30 mg	2	Schizophrenia (acute exacerbation)	Change from baseline at week 6 in the PANSS total score	Ongoing, estimated study completion June 2024
NCT03697252	Xanomeline 100–125 mg/Trospium 20–30 mg	2	Schizophrenia (acute exacerbation)	Change from baseline at week 5 in the PANSS total score	Significant difference of 11.6 PANSS total score points compared to placebo (20)
NCT04659161	Xanomeline 100–125 mg/Trospium 20–30 mg	3	Schizophrenia (acute exacerbation)	Change from baseline at week 5 in the PANSS total score	Significant difference of 9.6 PANSS total score points compared to placebo (21)
NCT04738123	Xanomeline 100–125 mg/Trospium 20–30 mg	3	Schizophrenia (acute exacerbation)	Change from baseline at week 5 in the PANSS total score	Ongoing, estimated study completion December 2022
NCT05145413	Xanomeline 50–125 mg/Trospium 20–30 mg additionally to the established antipsychotic treatment	3	Schizophrenia (with an inadequate response to current antipsychotic treatment)	Change from baseline at week 6 in the PANSS total score	Ongoing, estimated study completion March 2024

PANSS, Positive and negative syndrome scale.

These developments make it seem possible that in the near future, a second substance class of muscarinic antipsychotics may be established alongside current antidopaminergic antipsychotics. This would lend new urgent importance to the question of whether there is an “antimuscarinic subtype” in schizophrenia and, particularly, how to identify it in clinical settings since this subtype might show non-response to antidopaminergic medication and response to procholinergic treatment. This perspective is intended to present several potential *in vivo* markers of muscarinic deficit in schizophrenia that ideally in the future could be used for personalized therapy planning and help in deciding whether to initiate treatment primarily with a dopamine antagonist or a muscarinic acetylcholine agonist.

## 2. Possible biomarkers for muscarinic dysfunction in schizophrenia

The following sections describe markers that could indicate a muscarinic deficit in patients with schizophrenia. A summary is provided in Table 2.

**Table 2:** Possible markers of muscarinic deficits in schizophrenia and brief summary of the evidence.

**Marker**	**Summary of evidence**
Treatment resistance to dopamine antagonists	Circa 25% of schizophrenia patients do not respond to antidopaminergic antipsychotics, but, partly, to clozapine, whose main metabolite might act through M1 receptor agonism.
Visual hallucinations	Muscarinic antagonists frequently induce visual hallucinations. Visual hallucinations occur in a subset of schizophrenia patients. In some cases, visual hallucinations in schizophrenia patients can be treated with procholinergic interventions.
Marked cognitive deficits	Muscarinic antagonists frequently induce cognitive deficits and disorganized symptoms. Both occur to varying degrees in schizophrenia patients.
Reduced mismatch negativity	Mismatch negativity (the electrophysiological response to a rule violation in a sequence of stimuli) is reduced in schizophrenia patients and linked to cognitive deficits and treatment resistance to dopamine antagonists. Mismatch negativity is reduced by anticholinergic drugs and increased by procholinergic drugs in healthy individuals.
Presence of antimuscarinic antibodies	Anti M1 antibodies are more frequent in sera of schizophrenia patients compared to controls.
Reduced M1 receptor availability in SPECT scans	M1 receptor availability is reduced schizophrenia patients compared to controls.

### 2.1. Non-response to dopaminergic antipsychotics

About 20% of schizophrenia patients show no improvement at all when treated treated with common antipsychotics (23). Currently, clozapine is the most effective antipsychotic for patients with non-response to antidopaminergic agents (24). Its superiority over all other antipsychotics, as well as its particular efficacy (25) in non-responders to antidopaminergic antipsychotics hints to a unique non-dopaminergic mechanism of action of clozapine. In this context, it is crucial to note that N-desmethylclozapine, the major metabolite of clozapine, exhibits unique agonism at the M1 receptor (26, 27), potentially making it the first representative of the “procholinergic” antipsychotics (28). Its specific efficacy in non-responders to antidopaminergic antipsychotics together with the procholinergic property of clozapine's active metabolite make it conceivable that a proportion of patients unresponsive to antidopaminergic antipsychotics in fact have a muscarinic deficit underlying their symptoms, which is partially revised by N-desmethylclozapine. If this were true, the relative efficacy of other procholinergic antipsychotics such as xanomeline could be particularly high in patients with treatment resistance to antidopaminergic antipsychotics.

### 2.2. Specific pattern of symptoms

A hypothetical subtype of schizophrenia with a predominant underlying muscarinic deficit might show specific symptom patterns compared with other subtypes. Information about these clinical correlates of a muscarinic deficit may be provided by the symptoms elicited by anticholinergic agents. First and foremost here are cognitive impairments, which are caused by anticholinergics (29) and are also present in a large proportion of patients with schizophrenia (30). Moreover, intoxications with muscarine receptor antagonists induce psychotic states with specific characteristics which are only present in a minority of schizophrenia patients, like visual hallucinations and strong disorganization (31). Interestingly, case reports suggest that visual hallucinations in some schizophrenia patients can successfully be treated with acetylcholine esterase inhibitors, suggesting an underlying cholinergic deficit (32). From this, it can be hypothesized that patients with marked cognitive deficits and disorganization and with visual hallucinations might specifically benefit from procholinergic treatment.

### 2.3. Mismatch negativity

Mismatch negativity (MMN) refers to the electrophysiological response to a rule violation (e.g., an irregular pitch) in a sequence of (in most studies auditory) stimuli. A reduction of MMN in patients with schizophrenia compared to controls has now been shown in many studies and is considered one of the most promising biomarkers for predicting the risk of conversion to clinical psychosis in people at high risk and the response to specific therapies (33–35). In addition to the established deficits in higher cognitive processes, schizophrenia patients also show disturbances in basal sensory processes, such as pitch recognition (36). These sensory deficits are associated with difficulties in detecting linguistic subtleties such as irony or emotional coloring and consequently linked to the psychosocial functional impairments that are often prognostic in schizophrenia (36, 37). Measures of MMN are correlated to the magnitude of these sensory processing deficits (38). Consistent with that, recent studies revealed that reduced MMN is particularly present in a subgroup of patients with marked cognitive deficits and poor social functioning (34, 39, 40) and predicts poor response to therapy with common antipsychotics (41, 42). Studies on the neurophysiological substrate of MMN reductions have demonstrated associations to hypofunction of the glutamatergic NMDA receptor (43). However, a reduction in MMN through an antimuscarinic drug (scopolamine as compared to glycopyrrolate, a peripheral anticholinergic without effects in the CNS) has also been demonstrated in healthy individuals (44). Importantly, it was shown in a recent preregistered trial that only biperiden, a muscarinic acetylcholine receptor antagonist, but not amisulpride, a dopamine D2/D3 receptor antagonist, galantamine, an acetylcholinesterase inhibitor increasing acetylcholine levels, nor levodopa, a dopamine precursor increasing dopamine levels, changed mismatch negativity in healthy individuals (45, 46). Taken together, these findings suggest that muscarinic deficits entail reduced mismatch negativity, possibly through dysfunctional modulation of NMDA-dependent neurotransmission (4, 47). This would be consistent with the finding that response to antidopaminergic drugs is weaker in patients with strong MMN reductions. If reduced MMN indeed indicated a cholinergic deficit, it could be hypothesized that it predicts good response to procholinergic treatment.

### 2.4. Antimuscarinic antibodies

The exact pathomechanism underlying a potential cholinergic deficit in schizophrenia is unclear, with post-mortem studies suggesting reduced receptor density (15). Interestingly, antibodies targeting M1 receptors can also be found significantly more often in the sera of schizophrenia patients compared to controls, although this comparison ]based on few rather small studies (48). Within schizophrenia patients, one study reported significant correlations between M1 antibodies and specific negative symptoms (poverty of speech) (49), which are in turn predictive of poor outcomes under usual treatments (50). The exact effect of these antibodies on muscarinic receptors has yet to be elucidated and agonistic or antagonistic impacts are possible as well as an antibody-mediated destruction of receptors or receptor-expressing cells (48). Although data are still sparse, it seems possible that antimuscarinic antibodies play an etiologic role in a subset of patients. Their presence could accordingly be a predictive marker for a good response to promuscarinic treatment.

### 2.5. Neuroimaging

The muscarinic M1 receptor availability can be determined *in vivo* using radioactive tracers, such as 123I-IQNB for SPECT (51). In schizophrenia, a reduced M1 receptor availability compared to healthy controls was reported (52). With respect to associations between M1 receptor availability and specific schizophrenia symptoms, results have been mixed, with reports that lower receptor availability is related to increased positive symptoms (52) and, conversely, to increased cognitive deficits and negative symptoms but not positive symptoms (53). The sensitivity of *in vivo* assessments of M1 receptor status might be further increased with newly developed PET tracers for the M1 receptor such as 11C-LSN3172176 (54). Neuroimaging with radioligands thus has the potential to determine the extent of muscarinic deficit in schizophrenia and potentially to predict the benefit of procholinergic treatment. Along similar lines, the striatal dopamine synthesis capacity assessed *via* F-DOPA-PET has been used to objectify a hyperdopaminergic state in schizophrenia patients (55). Recent research indeed showed that absence of hyperdopaminergia according to F-DOPA-PET predicted subsequent non-response to antipsychotics (56). Neuroimaging markers could thus potentially inform therapy assignment by providing primary procholinergic medication to patients who lack an increase in striatal dopamine synthesis but have a reduction in muscarinic receptor availability.

## 3. Discussion

The current success of agonists at muscarinic acetylcholine receptors in the treatment of schizophrenia makes it seem possible that in the future a second substance class of procholinergic antipsychotics will be established alongside the primarily antidopaminergic antipsychotics. In this case, it would be important for individualized treatment to find specific predictors of response to procholinergic (compared with antidopaminergic) antipsychotics. Possible candidates for such predictors include treatment resistance to antidopaminergic treatment, the presence of severe cognitive impairment and/or visual hallucinations, reduced mismatch negativity, the presence of antimuscarinic antibodies, and reduced availability of muscarinic receptors on radionucleotide imaging.

Muscarinic acetylcholine receptors can be divided into 5 subtypes (M1–M5), with mainly agonists at M1 and M4 subtypes being considered candidates in schizophrenia treatment, whereas stimulation of M2 and M3 subtypes is related to gastrointestinal and other peripheral side effects. Animal research furthermore suggests that enforcing stimulation at M4 receptors through positive allosteric modulators (PAM) particularly improves proxy markers of positive schizophrenia symptoms such as hyperlocomotion or impaired prepulse inhibition induced by dopamine agonists or NMDA antagonists (28, 57). In contrast, PAM at M1 receptors have mainly been investigated for cognition-enhancing potentials across different neuropsychiatric disorders including schizophrenia. For instance, it could be shown that PAM at M1 receptors improve performance in tasks designed to assess cognitive ability (e.g. novel object recognition task) in schizophrenia mouse models (28, 57). While xanomeline is an M1 and M4 preferring but not fully specific muscarinic agonist, these results highlight that possibly in the future specific schizophrenia symptom clusters could be addressed with selective modulators. In this case, treatment could be guided by both predominant symptoms (positive vs. cognitive symptoms) and possibly by investigations with subtype-specific PET tracers (58).

A crucial question in attempts to find biomarkers for response to muscarinic agonists concerns the precise cause-effect relationship between muscarinic deficit and psychotic symptoms, specifically the question, if muscarinic deficiency causes psychosis through dopaminergic or dopamine-independent pathways. Obviously, the hope to identify distinct predictors for the response to antidopaminergic vs. procholinergic treatment rests on the assumption that there are dissociable etiologies behind psychotic syndromes (schematically, a hyperdopaminergic and a hypomuscarinergic etiology, respecetively). A variety of complex interactions between muscarinergic and dopaminergic neurotransmission have been established (59), leading to the hypothesis that muscarinic agonists ultimately work through modifying dopamine release (14). This hypothesis is supported for example by the finding that psychotic symptoms in M1 receptor knockout mice are related to increased dopamine levels in the nucleus accumbens (60). Conversely, substantial parts of the evidence summarized above like the existence of a distinguishable subgroup with marked deficiency in muscarinic receptors, the non-response to antidopaminergic drugs in a proportion of patients, and the discovery that non-responding patients show no alterations in the dopamine system according to PET studies speak for the existence of patient subgroup where psychotic symptoms are mainly caused by dopamine-independent mechanisms (3). Accordingly, it has been hypothesized that different subtypes of schizophrenia pathophysiologically involve a disturbance in the dopaminergic vs. cholinergic modulation of glutamate receptors, which ultimately causes the development of psychotic symptoms (4). This would indeed imply a primarily dopaminergic vs. a primarily cholinergic etiology, and thus the hope that (a proportion of) patients with inadequate response to dopaminergic interventions could benefit from muscarinergic agents. Fittingly, a potential synergism of antidopaminergic and promuscarinic interventions is suggested by animal studies showing enhanced efficacy of classical antipsychotics by additional administration of M1 receptor PAM (61). Results from the ongoing ARISE trial, which is evaluating the efficacy of xanomeline-trospium as add-on therapy in 400 patients with inadequate response to antidopaminergic antipsychotics such as risperone, paliperone, and aripiprazole and is scheduled to be completed in March 2024 (62), may soon provide new insights into this debate: Adding a second antipsychotic to a first antipsychotic has generally not been shown to be effective in randomized trials (63) and is not a recommended strategy in the case of non-response (64), which may be because all current antipsychotics ultimately act through dopaminergic mechanisms and therefore no synergistic effect occurs when combining multiple agents. Hence, xanomeline proving effective as an add-on treatment would be (tentative) evidence that it indeed possesses a distinct non-dopaminergic mechanism of action.

The most rigorous way of testing the capacity of the suggested biomarkers to identify those schizophrenia patients who will likely benefit more from a procholinergic as compared to an antidopaminergic treatment would be to test their specific ability to predict individual outcome of a clinical trial of, for example xanomeline-trospium compared with amisulpride treatment. A specific predictor, as compared to a general prognostic factor, is a pretreatment variable (e.g., a quantification of the markers listed above), whose influence on the treatment outcome (e.g., on the reduction in a schizophrenia severity score such as the positive and negative syndrome scale) differs between treatments (65). For instance, it could be hypothesized that presence of antimuscarinic antibodies predicts good treatment outcome in the xanomeline arm, but bad outcome in the amisulpride arm of a trial. Statistically, this would be captured in a significant interaction effect between presence of antimuscarinic antibodies and treatment in a predictive model (65). A study testing such treatment-predictor interactions would be quite effortful, as all potential markers would need to be collected at baseline and the detection of treatment-by-variable interactions requires relatively large sample sizes (66). However, if successful, i.e., if one or more markers prove to be specific predictors of the outcome of procholinergic or antidopaminergic treatment, it would represent a real milestone in the individualized treatment of schizophrenia.

## Data availability statement

The original contributions presented in the study are included in the article/supplementary material, further inquiries can be directed to the corresponding author.

## Author contributions

The author confirms being the sole contributor of this work and has approved it for publication.
